# Unveiling the Impact of Maternal Hyperthermia in the Late First Trimester: A Case Report of Anterior Esthetic Rehabilitation Utilizing Heterodontic Biologic Posts

**DOI:** 10.7759/cureus.64922

**Published:** 2024-07-19

**Authors:** Sriparna De, Anshul Gangwar

**Affiliations:** 1 Pediatric Dentistry, Institute of Dental Sciences, Bareilly, Bareilly, IND

**Keywords:** case report, genetics, pregnancy, fever, biologic post

## Abstract

The perinatal maternal environment is important for the normal development of the fetus. Epigenetic modifications that influence developmental control genes and signalling pathways for proper fetal development have been associated with maternal illnesses brought on by viruses, bacteria, or even parasitic protozoa. It is crucial to provide details on the onset, length, and timing of the mother's fever because these factors may influence the kind of certain abnormalities. Although fever is a primarily benign disease, it has been linked to negative health outcomes in children and has occasionally resulted in a substantial referral to critical care. This case report presents a 15-year-old female patient with repaired cleft palate and tetralogy of Fallot (TOF) who approached for esthetic rehabilitation of lower anterior teeth. The teeth (31, 32, 43) were tender on percussion. Radiographic evaluation showed the presence of periapical radiolucency. The root canal procedure was performed under local anaesthesia, and the supernumerary maxillary teeth were extracted. After cleaning and disinfecting, these teeth were used as biologic posts with respect to 32 and 33. A follow-up examination was performed after 12 months. The results of this case indicate that using autologous heterodontic biologic posts can lead to a favourable outcome.

## Introduction

Fever during pregnancy is common, with approximately 20% of women reporting at least one febrile episode [1]. Fever related to urinary tract infection (UTI) was found to be the third most frequent cause. Although there were no maternal deaths, there were unfavourable fetal outcomes, such as preterm deliveries and low birth weight newborns. This indicates the necessity of increasing the use of bacterial sensitivity-guided antibiotic usage in pregnant women with UTIs and of urine cultures as a screening tool for asymptomatic bacteriuria [2]. Pregnant women have a reported 8% incidence of UTI [3]. Despite being a relatively benign condition, fever was associated with several neonatal affections and adverse health affections in offspring, leading sometimes to a significant referral to intensive care [1].

The development of a functioning circulatory system is necessary for the embryo to survive, and it starts as early as 28 days after conception [4]. The most prevalent cyanotic congenital heart condition, tetralogy of Fallot (TOF), affects around 1 in 3600 births and accounts for 7-10% of all congenital cardiac abnormalities [5]. Palatogenesis begins in the sixth week and is completed by palatal fusion at 12 weeks of gestation [6]. Failure to fusion of palatal shelves leads to CP and has a high prevalence of dental anomalies, such as dental agenesis, supernumerary teeth and morphologic irregularities [7]. The mandibular second premolar (3.4%) and maxillary lateral incisor (2.2%) are the most often congenitally missing permanent teeth, excluding third molars. A survey on congenitally missing teeth revealed that the incidence of congenitally missing permanent canines was 0.1% in the maxilla and 0.02% in the mandible [8]. We emphasize that the present case with dental anomalies may be because of partial expression of PAX and MSX gene family due to pathogenic infection during pregnancy. After one year of successful dental treatment, we could not find any recurrence of the lesion. The patient was asymptomatic and the teeth were functional.

## Case presentation

**Figure 1:**
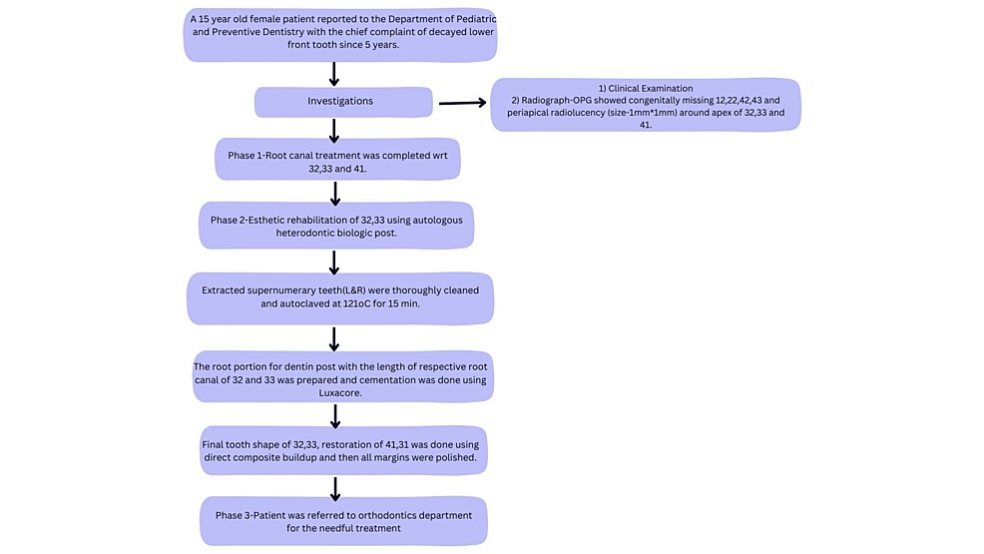
Sequential timeline diagram emphasizes the events and their depiction

A 15-year-old female patient (student of class X) reported to the Department of Pediatric and Preventive Dentistry with a chief complaint of a decayed lower front tooth for five years (Figure 1).

**Figure 2:**
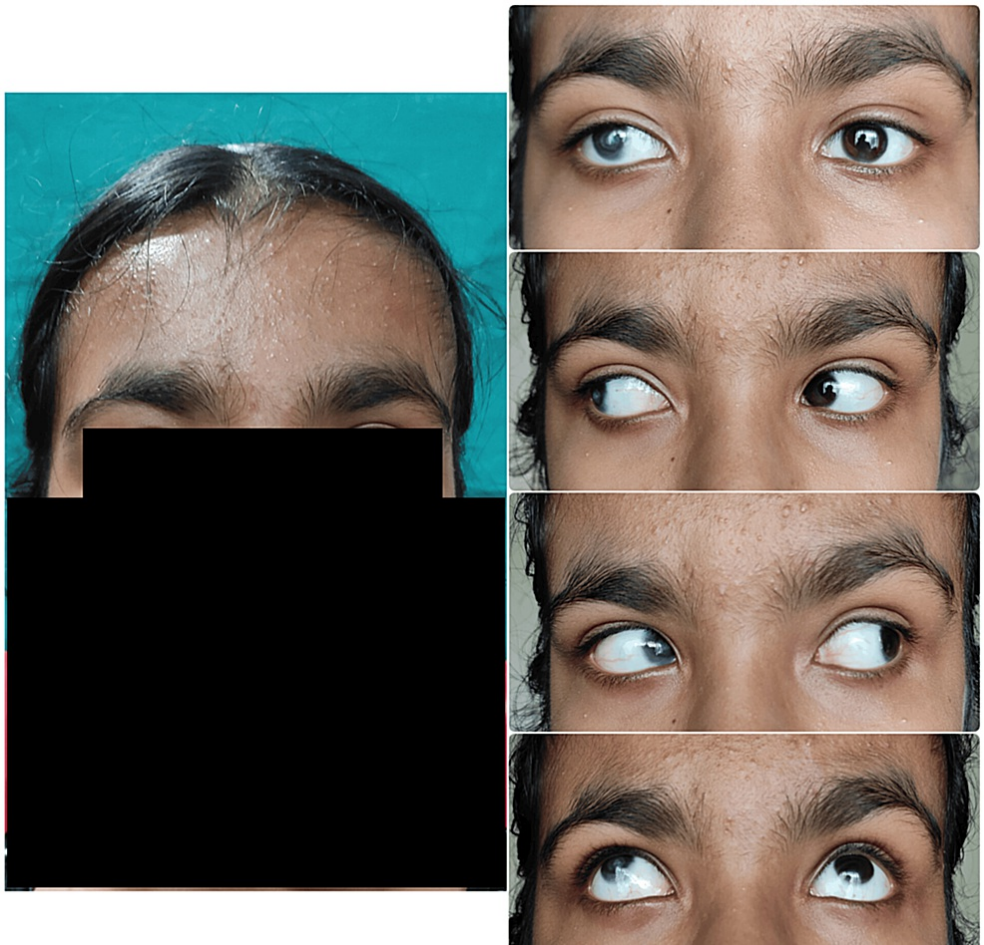
Extraoral examination of the patient showing bilateral corneal opacity

On extraoral examination, the patient had a broad forehead with a high hairline and sparse hair. She had bilateral corneal opacity (Figure 2).

**Figure 3:**
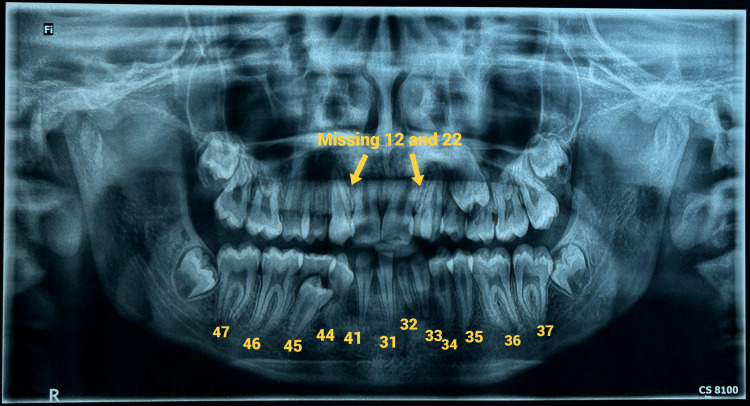
Pretreatment panoramic radiograph

Intraoral examination revealed a repaired cleft palate with clinically missing teeth 12 and 22, a crossbite with respect to tooth 13, a rotated tooth 23, a scissor bite with respect to tooth 26, and labially placed bilateral maxillary supernumerary teeth with a shift in the midline. She also had difficulty pronouncing words starting with "s" due to carious mandibular anterior teeth. The radiograph (orthopantomogram, or OPG) revealed a full set of permanent teeth with congenitally missing teeth 12, 22, 42, and 43, as well as periapical radiolucency (size: 1 mm x 1 mm) around the apex of teeth 32, 33, and 41 (Figure 3).

Medical history

On detailed enquiring about medical history, the mother revealed that during the late first trimester, she suffered from high-grade fever with UTI. During a routine ultrasound scan in the early second trimester by a gynaecologist and ultrasonologist, the parents were asked to abort the child (fetal age: 16 weeks) due to fetal abnormality, but the parents denied it because she was their first child and they were ready to take the risk. The preterm (7.5 weeks) child was delivered by C-section with bilateral corneal opacity, TOF and cleft palate. At the age of 10 months, there was a diminution of vision (the right eye was more affected than the left eye). The ophthalmologist advised cornea transplantation but parents did not opt for surgery due to financial difficulties. The child was operated on at the age of 18 months for TOF and cleft palate repair at two years of age. A proper medical record of previous investigation and treatment could not be obtained as they were not properly maintained. Parents were recommended to undergo karyotyping; however, due to financial constraints, they were unable to proceed with the procedure.

The patient was presented with all available treatment options for teeth 32, 33, and 43 including extraction and replacement, root canal treatment followed by biological posts and restoration. The pros and cons of each option were explained in detail. The second option was ultimately chosen due to the patient’s desire and obtained written informed consent from the patient.

**Figure 4:**
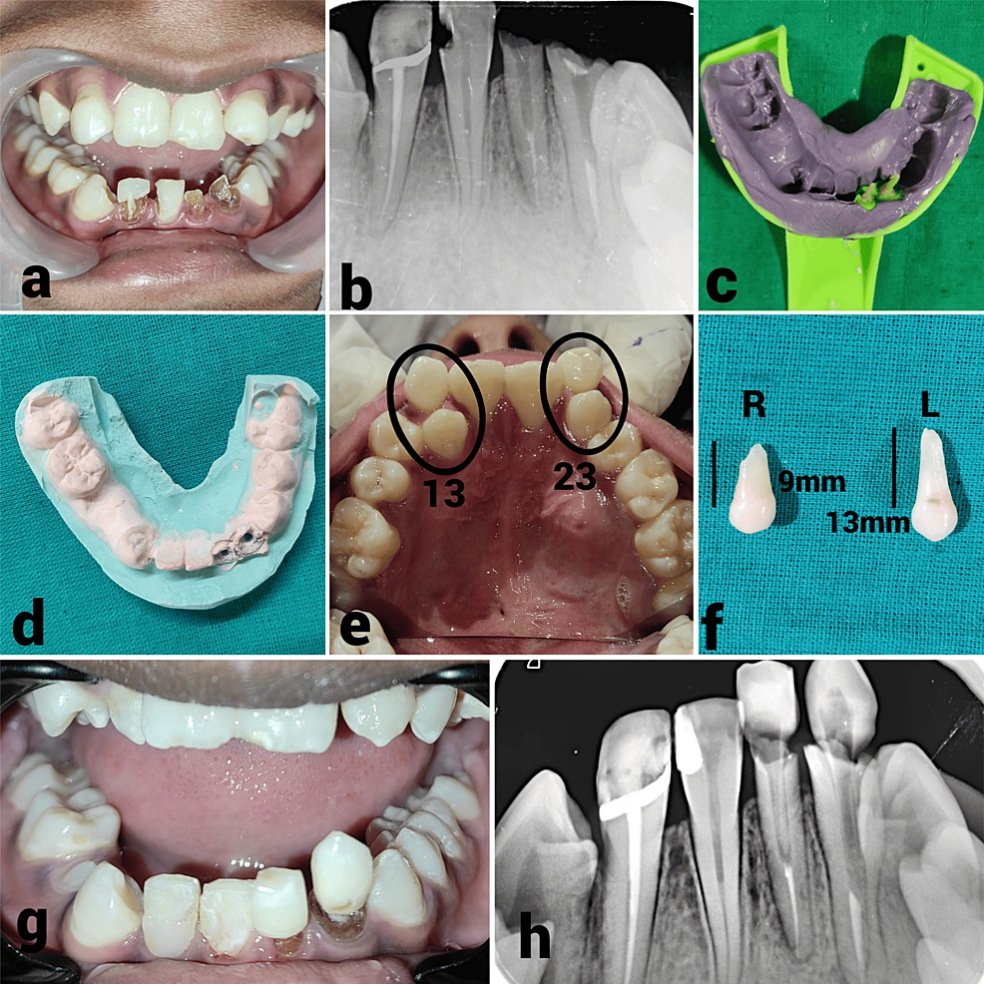
Sequential stages of endodontic intervention of lower anterior teeth (a) Preoperative photograph revealed grossly decayed teeth 32, 33, and 41; (b) Obturation of teeth 32, 33, and 41 done using gutta-percha and coated with AHPlus sealer (Dentsply Sirona, York, USA); (c) Impression of the post space of teeth 32 and 33 made with a custom-made self-cure post and medium-bodied elastomeric impression material; (d) Impression was poured with die stone to create a model, which served as a guide during the fabrication of the dentin post; (e) Extraction of both maxillary supernumerary teeth (left and right) was performed; (f) Extracted teeth were thoroughly cleaned and autoclaved at 121°C for 15 minutes; (g) Each tooth was trimmed circumferentially along the root portion to match the length of the respective root canal of teeth 32 and 33; (h) The model served as a reference for orienting the shape, length, and thickness of the dentin post, which was radiographically verified.

The treatment plan was done phasewise. In phase 1, root canal treatment was completed with respect to teeth 32, 33, and 41 after nonsurgical healing of periapical lesions. In phase 2, esthetic rehabilitation of teeth 32 and 33 was done using autologous heterodontic biologic post. For dentin post and core fabrication, the extracted supernumerary teeth (right and left) were thoroughly cleaned and autoclaved at 121 degrees Celsius for 15 minutes. The root portion for the dentin posts, matching the length of the respective root canals of teeth 32 and 33, was prepared, and cementation was performed using LuxaCore (DMG America, Englewood, USA). The final tooth shape of teeth 32 and 33 and the restoration of tooth 41 were accomplished using direct composite buildup, followed by polishing all margins (Figure 4).

**Figure 5:**
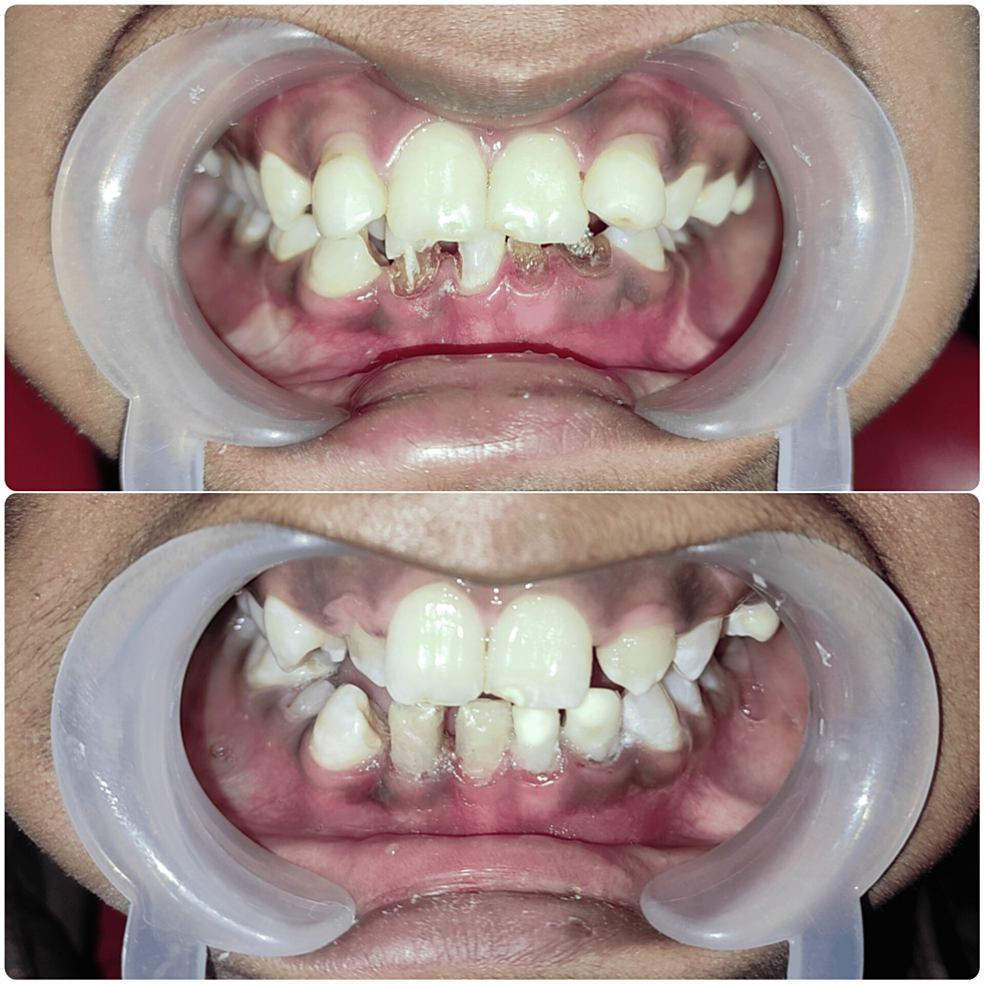
Before and after treatment photographs

In phase 3 (future prospective plan), the patient was referred to the orthodontics department for necessary treatment. However, since the patient was in class 10 and appearing for board exams, her parents decided to defer further orthodontic treatment at this time. The patient was recalled for follow-ups at the end of three, six, and 12 months. The patient remained asymptomatic during the three-month, six-month, and 12-month follow-ups. On clinical examination, the teeth were nontender on percussion (Figure 5). The patient was satisfied with the treatment.

## Discussion

Cellular processes and molecular events are highly regulated throughout organogenesis. The fetal immune system is incompetent and more vulnerable to infections spread vertically. Changes in the ratio of pro- to anti-inflammatory genes have been seen in abnormal inflammatory cytokine release as a result of maternal infection [9].

The PAX gene family, also known as the paired box (PAX) genes, codes for certain DNA-binding transcription factors that usually have a paired-type homeodomain and a PAX domain. There are nine genes in the mammalian PAX gene family that encode transcriptional regulatory proteins that bind DNA [10]. PAX gene mutations have been related to a variety of fetal abnormalities. All members of the PAX gene family influence cell fate, apoptosis, and differentiation, which all contribute to the formation of organs during embryogenesis [11].

One of the transcription factor family's genes, PAX9, is found on chromosome 14 (14q12-q13). These genes are crucial for the early stages of development in a number of multicellular animals [12]. PAX9 is essential for the development of the cardiovascular system because it causes a variety of cardiovascular abnormalities in newborns and embryos. In people with cardiovascular abnormalities and other disorders, gene mutations and chromosomal deletions, including PAX9, have been found [13]. TOF is also linked to new potential candidate genes, including CLDN9, CCDC168, SN2, L1TD, TACC3, CCDC36, and TTC5 [14]. The homeobox gene locus PAX6 is responsible for the transcription of several genes and growth factors involved in the development of the eye. Cells of the eye field express a set of eye field transcription factors (EFTFs) that are highly conserved throughout vertebrates. In mammals, the EFTFs include PAX6, RAX, SIX3, and LHX2 [15]. The transcription factors, PAX7 and PAX9, control the growth and differentiation of cranial neural crest cells, which in turn affects the creation of the craniofacial area. The interruption of cranial neural crest cell development resulting from dysfunction and impairment causes craniofacial deformities, such as orofacial clefts [16].

The genetic processes controlling the development of the human dentition are revealed via heritable tooth agenesis. The incidence of spontaneous agenesis, which often results in one to three missing teeth, may be impacted by hereditary or local circumstances. Based on cellular, molecular and genetic studies, genes PAX9, MSX1, and Bmp4 (bone morphogenetic protein-4) are responsible for tooth development. Oligodontia and hypodontia have been linked to mutation of PAX9 and MSX1 gene. Tooth agenesis is associated with several genes; however, non-syndromic human tooth agenesis has been associated with mutations in AXIN2, EDA, PAX9, MSX1, and PAX9 [17]. Unusual tooth agenesis is represented by nonsyndromic agenesis of permanent mandibular canines. Despite the fact that no particular genetic pattern or aberration has been linked to the agenesis of a single permanent lower canine.

A sporadic case is characterised by a novel mutation that arises spontaneously for no apparent cause in individuals without a positive family history of the condition. Since there was no positive family history, the present case is an illustration of a sporadic mutation. The patient suffered from a mild form of TOF associated with bilateral corneal opacity, incomplete cleft palate, bilateral maxillary supernumerary teeth and agenesis of teeth 12,22,42,43. Based on the literature and clinical findings of the present case, it can be suggested that there might be a case of mutation in the PAX and MSX gene family (Tables 1-2).

**Table 1:** Genes associated with tooth agenesis in humans Reference: [18]

Genes involved	Mutations of genes	Defect
PAX9	K114X, L21P, R26W, R28P, G51S, K91E, G73fsX316, V265fsX316 & R59fsX177	Molar hypodontia, oligodontia, peg-shaped laterals
MSX1	M61K, S105X, Q187X, R196P & S202X	Hypodontia, oligodontia
AXIN2	Arg656Stop, 1994- 1995insG	Incisor agenesis
LTBP3	Y774X	Oligodontia
EDA	Thr338Met	Hypodontia

**Table 2:** Developmental roles of PAX genes on different human tissue types Reference: [19]

	Developmental role on human tissue types	Master regulator gene
Group I	Teeth	PAX 9
	Thymus, parathyroid	PAX 1
	Skeleton	PAX 1, PAX9
Group II	Thyroid	PAX 8
	B-cells	PAX 5
	Kidneys	PAX 2, PAX 8
Group III	Skeletal muscle	PAX 3, PAX 7
	Neural crest	PAX 3, PAX 7
Group IV	Eye	PAX 6
	GI endocrine cells	PAX 4, PAX 6
	Central nervous system	PAX 2, PAX 3, PAX 5, PAX 6, PAX 7, PAX 8

A phenotype-driven molecular genetic test was recommended to rule out genetic abnormality. Congenital abnormalities rank among the top 20 worldwide sources of illness burden. It is known that pregnancy-related hyperthermia is teratogenic. When protein synthesis is disrupted by hyperthermia, cell death, membrane and vascular disruption and placental infarction may result. This may result in fatalities or serious fetal deformities. As the first trimester of pregnancy is when any teratogenic influence on the fetus is most likely to occur, we concentrated on investigating the relationship between fever in pregnancy and fetal congenital abnormalities.

## Conclusions

Cytogenetic testing provides an opportunity to enhance the prevention of congenital disorders. Parents can receive counselling regarding expectations for a specific congenital disorder, assess fetal and maternal risks, and make informed decisions regarding pregnancy continuation. The risks associated with terminating a pregnancy increase without an early cytogenetic diagnosis. Our data suggests that fever itself or other physiological changes linked to numerous infections are correlated with certain birth defects. Women who are pregnant or planning to become pregnant may want to consider speaking with their healthcare provider about the best ways to avoid infections that may cause fever and guidance on how to treat fevers during pregnancy.
